# Effects of tattoos on the aesthetic appreciation of human stimuli as influenced by expertise, tattoo status, and age reflecting internalized social norms

**DOI:** 10.1371/journal.pone.0313940

**Published:** 2024-12-11

**Authors:** Selina M. Weiler, Christian Duer, Dustin Krämer, Thomas Jacobsen

**Affiliations:** Experimental Psychology Unit, Humanities and Social Sciences, Helmut Schmidt University / University of the Federal Armed Forces Hamburg, Hamburg, Germany; University of Maiduguri, NIGERIA

## Abstract

Scientific interest in body modifications continues to grow, and tattoos have recently become a subject of empirical aesthetics. While conceptual structures of tattoo aesthetics have been studied, the question of how tattoos are aesthetically appreciated has not yet been studied. In this study, we examined how tattoos influence the aesthetic appreciation of human stimuli and uncovered differences in beauty perceived by individuals older and younger than 50, which we consider indicative of different internalized social norms, experts (tattoo artists) and nonexperts, and tattooed and nontattooed individuals. Images of a male and a female model were manipulated to vary in the amount of tattoo coverage across six manipulation conditions: Baseline (none), Light, Moderate, Heavy, Extreme, and Extreme + Face. *N* = 487 participants rated the beauty of these stimuli. The results suggest overall group differences (experts vs. nonexperts; tattooed vs. nontattooed; older vs. younger). The perceived beauty of the stimuli decreased as the extent of tattoos increased, with the Extreme + Face condition standing out as the lowest rated condition. These findings confirm that tattoos influence aesthetic appreciation, which is highly dependent on expertise and social norms as indicated by age. We also discuss the generalizability and implications of the findings.

## Introduction

Tattoos involve (semi-)permanent selective puncturing of the skin with needles and injecting color into the skin’s middle layer [1]. This phenomenon has been observed across cultures for millennia [2, 3] and, taking different age groups and social group affiliations into account, is estimated to characterize 3% to 25% of the world’s population [4–9]. Tattoos hold a deep cultural significance, which this study aims to explore beyond just their historical and social contexts by focusing on how people aesthetically appreciate them. We’re particularly interested in understanding how different factors—such as a person’s level of expertise in tattoos, whether they have tattoos themselves, and their age, as a proxy for social norms—might affect the way they perceive images of tattooed individuals. The reasons for acquiring a tattoo are at least as multifaceted as the motifs and styles of the tattoos themselves [3, 10]. Tattoos have deep mental functions for their wearers, representing more to them than mere fashion [3, 11]. The recent Review of Weiler et al. [11] highlight the complex psychological functions of tattoos, including their role in aesthetic perception and group identity. These findings are of particular interest in the context of research on mating choices in evolutionary psychology [e.g., 12]: Galbarczyk and Ziomkiewicz [13] have shown that tattoos can increase the perceived physical attractiveness of a male stimulus, but the mechanisms underlying the aesthetic processing of this stimulus remain unexamined. However, prior research suggests that perceived physical attractiveness may be influenced by internalized social norms and expertise [13].

Tattoos, once marginalized in early 20th-century Western cultures [3], saw a resurgence in popularity from the late 1950s, often symbolizing rebellion and individuality, particularly among subgroups such as bikers [14, 15]. This trend towards increasing acceptance continued into the 1990s, described as a second renaissance by DeMello [14], leading to tattoos becoming widely accepted in some social classes [14, 16–22]. While tattoos have reached popularity in the Western world, their acceptance varies globally, with some non-Western cultures maintaining restrictive views: South Korea, for instance, has only recently begun to soften its stance on tattoos, still requiring them to be done by certified physicians [23].

At the same time, even in the Western world, tattooed individuals continue to experience discrimination, stigmatization, repression, and even bans [15, 24, 25]. In 2020, the European Union decided to ban the use of numerous tattoo inks due to the health risks posed by specific ink ingredients [26], and this ban went into effect at the beginning of 2022 [26]. Many tattooed individuals face discrimination in their personal and professional lives—for instance, they may have lower chances of being hired and are more likely to be judged negatively in job interviews [27]. Tattooed individuals are particularly disadvantaged in occupations characterized by intense customer contact and a need for trust [28]. As a consequence of discrimination and career-specific disadvantages, some individuals wear their tattoos in a hidden manner, or even decide against getting more tattoos [29]. Sometimes, to avoid further negative experiences, individuals even have their tattoos (partially) removed [30].

### Expertise

Regarding expertise, it has been found that experts generally differ from nonexperts in their preference for works of art with higher complexity [e.g., 31–34], that their emotional aesthetic judgments differ from those of nonexperts [e.g., 35], that the limbic systems of music experts [e.g., 36] differ from those of nonexperts [e.g., 37], and that experts find asymmetry more beautiful than nonexperts [e.g., 38]. In a first study on the conceptual structure of tattoo aesthetics, experts also differed from nonexperts in presenting a clear positivity bias and mentioning terms referring to aesthetic dimensions (e.g., beauty or artfulness) more frequently [39]. For the purposes of this study, the term “experts” is specifically defined in the Participants section. This study posits that expertise in tattooing—characterized by a nuanced appreciation for complexity and artistry—will correlate with higher aesthetic ratings for tattoos, reflecting a deeper understanding of the art form.

### Tattooed vs. Nontattooed

As early as 1997, Singh and Bronstad [40] had postulated that the addition or removal of tattoos alters a person’s perceived personality. Building on this basic assumption, the appreciation of tattooed individuals has become the subject of recent psychological research [12, 13, 41]. For example, [13] established an experimental paradigm in which participants were presented with stimuli consisting of photographs of male models with and without tattoos, edited using a raster graphics editor. Participants were then asked to rate the presented stimuli in terms of several attributes, such as personality traits [12, 13, 41]. In addition to personality traits, the influence of tattoos on perceived attractiveness has also been investigated [12, 13]. Male participants have been found to perceive male tattooed stimuli as more attractive, while female participants showed no difference in their appreciation of the same male tattooed and nontattooed stimuli [13]. Regarding different intensities of tattoos, no differences have been found in terms of perceived attractiveness [12]: Stimuli with large tattoos were not perceived as significantly more attractive than stimuli with medium-sized tattoos. Because of the partially conflicting results, it remains unclear whether tattoos increase or diminish aesthetic appreciation. Molloy and Wagstaff [12] suggested that the type, size, and position of the motif play a crucial role in appreciation and may thus affect the replicability of study results. Based on the influence of personality factors, we also explore whether individual differences, such as having tattoos, affect the aesthetic appreciation of tattoos, hypothesizing that tattooed individuals will have a more favorable view.

### Age as a proxy for social norms

The appreciation of tattooed individuals has been studied in various contexts over the last two decades [e.g., 13, 42]. Some studies have shown that individuals’ age, which is indicative of social norms, plays a role in their appreciation of tattoos [39, 43–45]. It has also been found that members of older generations are more critical and discriminatory towards tattooed individuals and rate them as less intelligent and sincere [43]. Young adults in the Western world are also influenced by tattoos [13, 41]. For instance, it has been shown that tattooed men are perceived as more attractive and more dominant, but also as worse partners and fathers [12, 13]. An overall tendency towards positive appreciation of tattoos among younger generations in the United States could carry over to subsequent generations [44]. Considering the impact of these social norm factors, our study hypothesizes that age (under 50 vs. over the age of 50), as a proxy for social norms, significantly influences aesthetic appreciation of tattoos, with younger individuals expected to rate tattooed stimuli more favorably.

### The present study

The interplay of social norms as reflected by age, expertise, and personal tattoo status, alongside the insights drawn from Weiler and Jacobsen [39] and studies highlighting the relationship between age and social norms [43–45], guides our investigation into the aesthetic appreciation of tattoos. This study aims to explore how these diverse perspectives influence the perception of beauty in tattooed human stimuli, hypothesizing that the stimuli will be rated as more beautiful by younger individuals (under the age of 50), experts, and those with tattoos. Our approach, which uses a clear age cutoff to explore the impact of societal acceptance, individual expertise, and personal identity with tattoos, further hypothesizes that neither the perspective from which the photographs are taken nor the sex of the model (pre-analysis) will significantly influence aesthetic appreciation, derived by previous research [e.g., 12, 46].

## Method

### Participants

**Fig 1:**
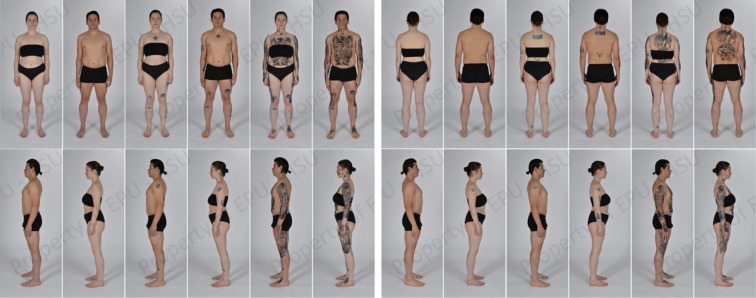
Male and female tattooed model seen in the four perspectives and six tattoo conditions. Exemplary stimuli for our male and female models in the Baseline, Light, Moderate, Heavy, Extreme, and Extreme + Face condition (from left to right), in the Ventral (upper left), Dorsal (upper right), Lateral Left (lower left), and Lateral Right (lower right) perspectives (Photos: Helmut Schmidt University / University of the Federal Armed Forces Hamburg, Creative Center, Ulrike Schröder).

We conducted a survey using the online survey portal *Unipark*, starting on 31.05.2022 and ending on 12.01.2023, and we employed IBM® *SPSS® Statistics* (Version 27) and *R* (version 4.2.3) software to carry out statistical analyses. A link to the survey was distributed via various channels: Helmut Schmidt University online bulletin, social media, flyers distributed in the lectures of the Helmut Schmidt University, and with the support of our research assistant D.K.. The data for the study were collected in two surveys (due to different recruiting methods; see below). For our statistical analyses, the participants in the two surveys were merged and considered separately (as participants over and under 50 years of age [the Age Group]; tattooed and non-tattooed individuals [the Tattooed Group]), and tattoo experts [the Expert Group]). There were no restrictions (other than the minimum age of 18) for participating in the study. However, we selectively recruited participants assigned to the Expert Group by explicitly contacting tattoo artists via e-mail; experts were defined as individuals with a minimum of seven years of experience as a tattoo artist. The research was granted ethical clearance by the Ethics Committee of psychology at the Faculty of Humanities and Social Sciences Helmut Schmidt University. It was executed in strict adherence to the tenets of the Declaration of Helsinki. The individuals pictured in Fig 1 have provided written informed consent (as outlined in PLOS consent form) to publish their images alongside the manuscript. Prior to any data acquisition, all participants provided their explicit written consent. Data gathering was conducted in a pseudoanonymized manner. Where applicable, participants were accorded course credit as compensation for their involvement. There was no monetary compensation for participation in the study, however, to increase the attractiveness of participating, a gift voucher worth 25 Euros was raffled among all of the participants.

Sociodemographic data and relevant variables for aesthetic appreciation and tattoos were collected, including age, gender, employment status, and sexual orientation [47], being tattooed, and how many tattoos each participant had.

Overall, *N =* 487 participants were recruited. Target sample sizes were determined a priori to ensure robust statistical power for the three four-way ANOVAs. An initial power analysis conducted using G*Power software [48] suggested a medium effect size of Cohen’s f (0.25; [49]) for each ANOVA, with an alpha level (α) set at 0.05 and a power (1 - ß) aimed at 0.80. This effect size was chosen based on its frequent use in empirical aesthetics research, reflecting the moderate but meaningful impact of aesthetic variables on preferences and responses. This analysis indicated a minimum requirement of 42 participants per group to reliably detect the anticipated effect sizes. Furthermore, using a sensitivity power analysis with our actual sample size of 487 participants, we established the capacity of our study to detect even smaller effect sizes, as minimal as dz = .07, maintaining the same levels of alpha and statistical power. This dual approach in our power analysis fortifies the confidence in our study’s findings, accounting for the four factors, their interactions, and the respective levels within each ANOVA. Additionally, as per Fritz and MacKinnon [50], detecting a medium mediation effect with 80% power typically requires a sample size of about 75 participants, given medium path effects between the predictor and mediator, and the mediator and the criterion. This further supports the robustness of our sample size. The participants were aged between 16 and 76 years (*M* = 33.39, *SD* = 12.81). For further details on sociodemographic characteristics, please see S1 Table.

### Experimental design and procedure

We employed a participatory quantitative research in this study, and conducted a cross-sectional survey to allow quantification of interindividual differences at a survey time point [51]. Our study adopted a within-subject experimental design, utilizing a 6 (Tattoo Condition) x 2 (Sex) x 4 (Perspective) factorial design to assess the aesthetic appreciation of tattoos. In more detail, the stimuli depicted either a male or a female model (Sex: Male vs. Female), viewed from one of four perspectives (Perspective: Ventral vs. Dorsal vs. Lateral Left vs. Lateral Right), in one of six conditions that varied regarding the extent to which the model was tattooed (Tattoo Condition: Baseline vs. Light vs. Moderate vs. Heavy vs. Extreme vs. Extreme + Face). As detailed in the Measures section, we excluded the dorsal view of the sixth level (Extreme + Face) from the experimental procedure due to its redundancy.

The participants were divided into groups based on their age (Age: Younger than 50 vs. Older than 50), whether they were tattoo experts (Expertise: Yes vs. No), and whether they were tattooed (Tattooed: Yes vs. No). The dependent variable, aesthetic appreciation, was measured through beauty ratings given by participants for each stimulus. The participants were presented with all stimuli in a randomized order and gave a beauty rating for each individual stimulus.

### Stimuli

Since the paradigm of presenting manipulated human stimuli [12, 13] has been proven successful, we used photographs of models in unobtrusive underwear and manipulated the extent to which they were tattooed. For this purpose, we used high-quality stick-on semi-permanent tattoos (from *Inkbox)* to create images that looked as realistic as possible. In a pre-study for tattoo selection, 16 scientific members of the Helmut Schmidt University were presented with all *Inkbox* tattoo designs that contained geometric shapes, nature motifs, and animal motifs, and were asked to rate them on a bipolar 7-point Likert-type scale ranging from −3 (*very negative*) to +3 (*very positive*). An average rating of greater than or equal to 1 was considered positive, and an average rating of less than or equal to −1 was considered negative [52]. Based on this pre-study, tattoo designs with ratings of +3 or -3 were excluded from the tattoo selection. Tattoos with writing and religious or political content were also deliberately excluded.

In contrast to the studies by Galbarczyk and Ziomkiewicz [13] and Molloy and Wagstaff [12], female stimuli were included. A 30-year-old male model and a 24-year-old female model, both without tattoos, were hired to be photographed. The models‘ heights were within Germany’s average range [53] and their body mass indices were within the normal range of the WHO body mass index recommendation of 18.5–24.9 [54]. A professional photographer took the photographs in a technical studio. The models were photographed in an upright, neutral posture, with nonsmiling facial expressions, from four different body perspectives: ventral, dorsal, lateral left, and lateral right. During the shoots, the models wore black, neutral underwear. The lighting and background were kept constant for all images. To ensure good adherence of the semi-permanent tattoos, the body hair (except the capillary hair) of both models was removed before the tattoos were applied. After the shoots, the photographs were edited by the photographer, and skin blemishes, lesions, and pigmentation were removed using a raster graphics editor. This resulted in a total of 46 stimuli (see Fig 1).

### Measures

For the main study, we generated items to survey the aesthetic appreciation of the presented stimuli. We operationalized aesthetic appreciation by the concept of beauty, because it has central importance for the experiential quality of an aesthetic stimulus and thus allows conclusions to be drawn about appreciation [52]. A bipolar 7-point Likert-type scale item *How beautiful do you find this person*?(Original instruction: *Wie schön finden Sie diese Person*?) followed the presentation of each stimulus, with the values ranging from *not at all beautiful* to *very beautiful*. (Original instruction: überhaupt nicht schön / sehr schön). For ethical reasons, we deliberately decided not to use an item with the wording *How ugly do you find this person*?

Given the stimulus features Tattoo Condition, Sex, and Perspective, our design would have initially resulted in a full 6 x 2 x 4 within-subject experimental design if all, of the photographs were presented to each participant of the study. However, we omitted the dorsal view of the sixth level of body modification—the tattooed face—since the face was not visible from the back and would have led to redundant stimuli, the identical dorsal views would have been presented twice. Noticing this stimulus repetition might have irritated participants, so we opted against it. Therefore, for the statistical analyses, we performed a value substitution for the sixth condition (Extreme + Face) by duplicating the values from the fifth into the sixth, as they represent the same stimulus picture. This adjustment was necessary to maintain a complete design for analysis, ensuring ecological validity. Inferential analyses were conducted three separate times—once for each division of the participant group based on the variables age, expertise, and tattoo status—with corrections applied to the alpha level to account for multiple comparisons.

Therefore, for the statistical analyses, we performed a value substitution for the sixth condition (Extreme + Face) by duplicating the values from the fifth condition into the sixth, as they represent the same stimulus picture. This adjustment was necessary to maintain a complete design for analysis, ensuring ecological validity. Inferential analyses were conducted three separate times—once for each participant group (age, expertise, and tattoo status)—with corrections applied to the alpha level to account for multiple comparisons.

### Data analysis

Statistical analyses were executed using R (Version 4.3.1, 2023-06-16; [55]) within RStudio (Version 2023.12.1+402; [56]). To ensure comprehensive data manipulation, visualization, and analysis, following packages were utilized: ez [57], tidyverse [58], ggplot2 [59], egg [60], afex [61], emmeans [62], haven [63], and sjlabelled [64] and CorelDRAW Graphics Suite 2022 for more advanced visualization [65].

Initially, the pre-analysis was conducted to ascertain if the perspective from which photographs were taken or the sex of the model influenced the aesthetic appreciation of tattoos, through three four-way ANOVAs focusing on Tattoo Condition, Perspective, Sex, and participant groups categorized by Age, Expertise, and Tattoo status. This step was crucial to confirm our hypothesis that these factors do not significantly affect the evaluation of tattoo aesthetics.

To examine the influence of age, expertise, and tattoo status on the aesthetic appreciation of tattoos, mixed ANOVAs with Greenhouse-Geisser corrections for each subgroup comparison were employed. This statistical approach was selected to manage potential violations of the sphericity assumption inherent in repeated measures designs. Post hoc comparisons were conducted using Tukey’s HSD tests to correct for multiple comparisons, allowing for an accurate identification of differences between conditions.

## Results

### Pre-analysis

To assure, in accordance with our preliminary hypotheses, that neither the perspective nor the sex of the stimuli had an influence of the aesthetic appreciation of tattoos, we conducted three four-way ANOVAs: Tattoo Condition x Perspective x Sex and participant group (Age, Expertise, and Tattooed). No significant evidence was found that contradicted our initial analyses: Expertise: *F*(12, 5820) = 1.53, *p* = .11; Tattooed: *F*(12, 5820) = 1.63, *p* = .08; and Age: *F*(12, 5820) = 0.99, *p* = .46.

### Hypotheses testing

**Fig 2:**
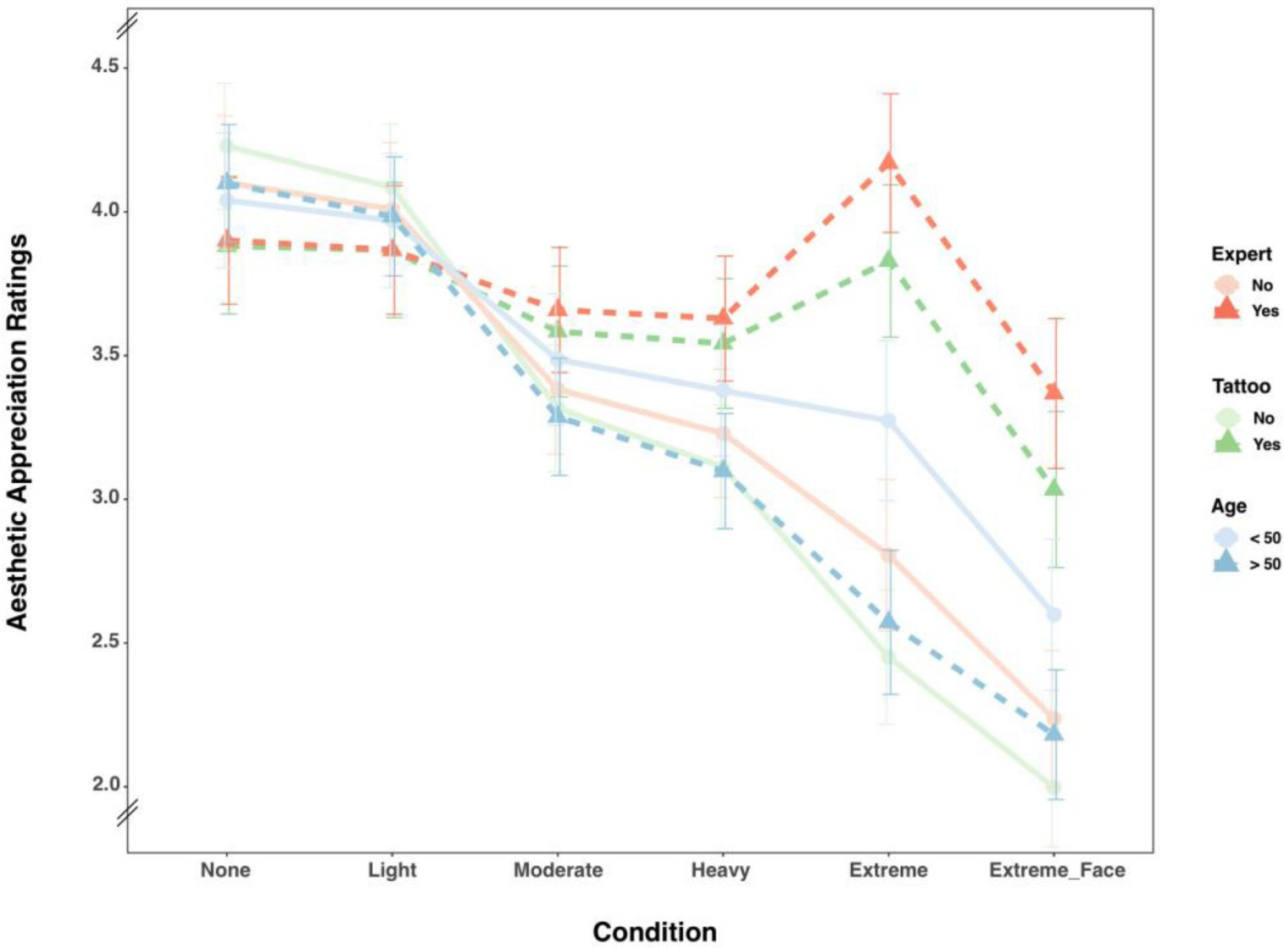
Aggregated rating data. Separate aggregations for the expert groups, tattoo groups, and age groups. Mean ratings shown separately for the six tattoo conditions and for three aggregations of the entire data: the expert groups, tattooed groups, and age groups. Note that the inference statistics cover the first five levels of the tattoo condition.

To investigate the influence of expertise (expert vs. non-expert), tattoo status (tattooed vs. non-tattooed), and age (under 50 vs. over 50) on the aesthetic appreciation of tattooed stimuli, mixed ANOVAs with Greenhouse-Geisser corrections were conducted for each main hypothesis, considering the varying degrees of tattoo coverage (Baseline, Light, Moderate, Heavy, Extreme, and Extreme + Face; see Fig 2).

### Expert vs. Nonexpert stimuli ratings

There was a statistically significant interaction between the Tattoo condition and Expertise, Greenhouse–Geisser *F*(5, 2425) = 76.70, *p* < .001, *η*^2^ = .14, large effect [49]. There were significant main effects for the Tattoo condition, Greenhouse–Geisser *F*(5, 2425) = 296.10, *p* < .001, *η^2^* = .38, large effect [49], and Expertise, Greenhouse-Geisser *F*(1, 485) = 26.80, *p* < .001, *η^2^* = .05, small to medium effect [49] (S2 Table).

For experts and nonexperts, the Baseline (*MDiff* = -0.20, 95%-CI[-0.02, 0.42], *p* = 1.00), Light (*Mdiff* = -0.14, 95%-CI[-0.08, 0.36], *p* = 1.00), and Moderate (*Mdiff* = 0.28, 95%-CI[-0.50, -0.05], *p* = .99) conditions did not differ significantly. The Heavy (*Mdiff* = 0.40, 95%-CI[-0.62, -0.18], *p* = .03), Extreme (*Mdiff* = -1.36, 95%-CI[-1.59, -1.14], *p* < .001), and Extreme + Face conditions (*Mdiff* = -1.13, 95%-CI[-1.35, -0.90], *p* < .001) differed significantly between experts and nonexperts (For more details, see S3 Table).

### Tattooed vs. Nontattooed stimuli ratings

There was a statistically significant interaction between the Tattoo condition and Tattoo status, *F*(5, 2425) = 117.39, *p* < .001, *η*^2^ = .08, medium effect [49]. There were significant main effects for the Tattoo status, *F*(1, 485) = 28.25, *p* < .001, *η*^2^ = .04, and the Tattoo condition, *F*(5, 2425) = 317.55, *p* < .001, *η*^2^ = .18, large effect [49] (S4 Table).

Between the tattooed and nontattooed groups, the Light (*MDiff* = 0.22, 95%-CI[0.02, 0.41], *p* = 1.00) and Moderate (*Mdiff* = -0.27, 95%-CI[-0.46, -0.07], *p* = .44) conditions did not differ significantly. However, the Baseline (*Mdiff* = 0.35, 95%-CI[0.15, 0.54], *p* = .03), Heavy (*Mdiff* = -0.43, 95%-CI[-0.62, 0.24], *p* < .01), Extreme (*Mdiff* = -1.38, 95%-CI[-1.60, -1.19], *p* < .001), and Extreme + Face conditions (*Mdiff* = -1.04, 95%-CI[-1.23, -0.84], *p* < .001) differed significantly for these groups (For more details, see S5 Table).

### Less than 50 vs. Over 50 years of age stimuli ratings

The results indicate a significant main effect for Age, *F*(1, 485) = 5.19, *p* = .023, with a small effect size [49], *η^2^* = 0.007, small effect [49], and condition Tattoo condition, *F*(5, 2425) = 260.39, *p* < .001, with a large effect size [49], *η^2^* = 0.17. Additionally, there was a significant interaction between the Age and Tattoo Condition, *F*(5, 2425) = 8.97, *p* < .001, with a small effect size [49], *η^2^* = 0.007 (S6 Table).

Due to significant main and interaction effects, we applied Tukey’s HSD-corrected post hoc tests. The Baseline (*MDiff* = -.06, 95%-CI[-0.34, 0.21], *p* = 1.00), Light (*MDiff* = -0.01, 95%-CI[-0.29, 0.26], *p* = 1.00), Moderate (*MDiff* = 0.20, 95%-CI[-0.08, 0.48], *p* = 1.00), Heavy (*MDiff* = 0.28, 95%-CI[0.004, 0.56], *p* = 1.00), and Extreme + Face (*MDiff* = 0.42, 95%-CI[-0.14, 0.69], *p* = .21) conditions did not differ significantly between the age groups. Only the Extreme condition (*MDiff* = 0.70, 95%-CI[0.43, 0.98], *p* < .001) differed significantly for the two groups (For more details, see S7 Table).

## Discussion

It seems that today, for several reasons, tattoos are becoming more and more accepted in Western societies [e.g., 3]. In the present study, we investigated how the perceived beauty of human stimuli is influenced by internalized social norms, expertise, and whether or not the perceiver is tattooed. For this investigation, we employed an established paradigm in which participants were presented with (and then asked to rate) tattoo-manipulated stimuli [in terms of the extent of the tattoos; 12, 13, 41]. Unlike in previous studies, however, the central evaluation criterion was not the presumed personality traits but rather the aesthetics of the stimuli. Here, aesthetics was operationalized by the construct of beauty [e.g., 51].

### Expertise

We assumed that experts would evaluate the tattooed stimuli as more aesthetically positive than would nonexperts. With regard to the conceptual structure of the aesthetics of tattoos [39, 66], differences between the groups have already been shown. Moreover, as Müller and colleagues [35] have reported, nonexperts frequently make aesthetic judgments based on internal affective responses, while experts rely more on an analytical approach when making judgments in the domain of their expertise. The results of the present study show that nonexperts and experts differ with respect to several levels of the manipulation. While the groups did not differ on the Baseline, Light, and Moderate conditions, experts appreciated the Heavy, Extreme, and Extreme + Face conditions more than those in the nonexperts group. We found the strongest difference in the Extreme condition: Experts not only assigned a higher rating to this condition than nonexperts, but also rated the stimuli in this condition higher than the stimuli in all other manipulation conditions. These findings imply that experts prefer individuals with full body tattoos (except for the face) over individuals without tattoos.

Numerous studies have already shown that experts indicate a preference for art with higher complexity [31–34]. Furthermore, Leder and colleagues [38] have shown that experts perceive asymmetric stimuli as more aesthetically positive than nonexperts. Given that tattooed bodies are perceived more asymmetrically [67] and that most of the tattoos that were placed on the models were based on predefined standards of placement, a general preference for asymmetric stimuli could partially explain the results. Nevertheless, the experts’ preference for extreme tattoos also seems to have limits. For instance, the Extreme + Face condition received the least positive rating among the experts. This suggests that experts show a preference for extreme tattoos but not necessarily for facial tattoos, which could be explained by the controversy surrounding facial tattoos, which apparently exists even among experts [68].

Because most of the experts in our study were tattooed (98.4%), the findings on ingroup biases [69, 70] can also be applied to our study: The mere-exposure effect [e.g., see also the discussion section in 39, 70] as well as attitudes [e.g., 65] could partially explain the results. The individuals’ personalities could also have affected experts‘ aesthetic appreciation of the stimuli, as tattooed individuals have been shown to have a greater need for uniqueness then nontattooed individuals [71–73], and the need for uniqueness has been found to be negatively related to the objective aesthetic goodness of figural compositions as measured by the VAST [Visual Aesthetic Sensitivity Test; 72]. Furthermore, tattooed individuals have more positive attitudes regarding tattoos [74], and lower needs for social conformity [75], which may have played a role in the predominantly tattooed expert group. Finally, simply being an expert and being surrounded by tattoos may have positively influenced ratings through the mere-exposure effect.

The *Body* in Jacobsen’s [66] *Framework for the Psychology of Aesthetics* suggests that individual differences in aesthetic appreciation may also be influenced by one’s biological preconditions. This is in line with the principles of psychophysics, which posit that sensory experiences are influenced by both physical stimuli and individual perceptual systems [76]. Thus, group differences in aesthetic ratings between tattoo experts and nonexperts may be due to differences in their perceptual systems. Neuroscientific studies have highlighted differences between experts and nonexperts on a neural level, particularly in structures of the limbic system [36]. Due to their intense exposure to specific entities such as tattoos, experts may benefit from processes of neuroplasticity, leading to more complex neural structures in areas identified as neurobiological correlates of the entity in question [77]. As a result, tattoo experts may experience more intense and positive aesthetic experiences than nonexperts due to the enhanced processing and integration of tattoo-related information in their perceptual systems.

Experts and nonexperts did not differ in their ratings between the Baseline and Light tattoo conditions. This suggests that nonexperts show some acceptance of small and concealable tattoos, which, given that all participants in this study were living in Germany, may be due to the generally increasing acceptance and popularity of tattoos in Western society [14, 16–19, 78].

### Tattooed vs. Nontattooed

When comparing the tattooed and nontattooed, it became apparent that the groups differed in their beauty ratings for the Baseline, Heavy, Extreme, and Extreme + Face conditions. As mentioned above, the more tattooed a model was, the higher the beauty rating by the tattooed individuals. While this effect was to be expected, it is interesting that tattooed and nontattooed individuals differed in their ratings for the Baseline condition: Nontattooed individuals rated these stimuli higher than tattooed individuals. Further, in both groups, individuals’ ratings did not differ between the Baseline and Light conditions, nor between the Moderate and Heavy conditions. Regarding the expert group, several points of view apply; while mere exposure [39, 79] and in-group bias [69, 70] also apply here, attitudes toward tattoos could also partially explain the effect. Since attitudes have an influence on aesthetic evaluation [66], the tattooed individuals might have had a more positive attitude towards tattoos than the individuals without tattoos, as suggested by the results. Because tattooed individuals modify their bodies for aesthetic reasons [3], we assumed that they would generally appreciate tattoos more than nontattooed individuals. This assumption has previously been confirmed by results on the conceptual structure of tattoos [39], in which more positive mental concepts regarding tattoos were identified by tattooed participants. This suggests that tattooed individuals might have exhibited cognitive schemas in which tattoos, and aesthetics are linked.

### Age as a proxy for social norms

Interestingly, the comparison of the groups of participants over and under the age of 50 did not follow the above pattern. The only difference between these groups was found in the Extreme condition, where the younger participants rated the stimuli as more beautiful than did the older participants. This finding is also consistent with previous research by Weiler and Jacobsen [39], who showed that age influenced the conceptual structure of tattoo aesthetics. In that study, individuals younger than 50 most frequently mentioned positive adjectives in a timed free listing task, whereas participants older than 50 most frequently used negatively associated adjectives to describe tattoos. However, the results of the present study only suggest that when the tattoos reached a certain extent, the younger group appreciated them more than the older group (except for the Extreme + Face condition). This age-related difference could be explained by numerous factors. The ubiquity of tattoos in the world of younger individuals may have led to a higher appreciation, as explained by the mere-exposure paradigm [79], both digitally [80] and in the real world [81]. More specifically, younger individuals have a significantly higher average social media use time [82], and might therefore be exposed to tattoos more frequently due to receiving personalized advertising content featuring tattooed individuals [83, 84]. In addition, many public figures—who frequently act as role models for younger individuals—feature tattoos in movies or advertising or have tattoos themselves [85–87]. This might trigger the desirableness [88] of tattoos among younger generations. Generally, not only public figures but also families and acquaintances may influence younger generations [39, 81]. The Contact Hypothesis [89], which states that negative attitudes as well as stereotypes can be reduced by more intense contact with distant groups, might explain the results as well. Similarly, previous studies have demonstrated the presence of negative stereotypes and attitudes in older individuals toward tattooed individuals [39, 43, 44, 90]. It is assumed that a person’s education has an influence on tattoo appreciation, since modern educational models primarily teach adolescents core traits such as tolerance and openness [39, 91, 92]. Studies have suggested that individuals with higher social tolerance and openness to experiences also have a higher tolerance regarding tattoos [74, 93–95]. Phenomena such as mental categorization processes could also explain the group differences here [69, 70]. It should be noted that the tattooed models that were rated in the present study were 24 and 30 years old. Since the age of the stimuli was not manipulated, no conclusions can be drawn about the variance in appreciation of younger versus older individuals.

Even though tattoos have increased in popularity, the aesthetics of the human body in its natural form (i.e., the Baseline condition) seems to be more appreciated, as seen in the mean ratings overall. An explanation of this preference for untattooed skin may be offered by symmetry as a central criterion for the aesthetics of entities [38, 66]. Specifically, tattoos could distort the symmetrical appearance of the human body and thus lead to reduced aesthetic appreciation. In this context, Osu et al. [67] showed that the number of tattoos on female bodies was positively related to their perceived asymmetry. Prevailing beauty ideals are defined by the aesthetic evaluations of the population at large [96–99]. The results of the present study could indicate that tattoos, which could now be perceived as mainstream, have survived the height of their fascination. This fascination might decrease in the future, especially among younger generations, since tattoos are no longer considered special, due to their omnipresence [1]. In other words, although they were once considered disreputable and exceptional, tattoos may have lost their appeal due to widespread social acceptance [9]. This assumption can be integrated into Jacobsen’s [66] model of the psychology of aesthetics, which postulates that aesthetic preferences change over time as reflected by the facet of *diachronia*. Given our results, we assume that trends in the fashion world change the aesthetic appreciation of some entities. Since acquiring and wearing tattoos provides a fashion statement [3], body modifications might lose their „hype” in the medium or long term.

### Limits of extreme tattoo appreciation

For all participants, the Extreme + Face condition exhibited a drastic decrease in aesthetic appreciation. A study by Zestcott et al. [68] offers an explanation: The researchers showed that participants exhibited negative implicit attitudes toward individuals with tattoos in the facial area. Since tattoos on the neck were used in the Extreme condition and tattoos on the face were used in the Extreme + Face condition, it can be assumed that the beauty rating of the stimuli was influenced by negative implicit attitudes toward facial (and in some cases near-facial) tattoos. Zestcott et al. [68] showed that the characteristics of tattoos have an effect on their appreciation: Negative implicit appreciation was attenuated when the motif of a tattoo was a positive symbol. Similarly, Jacobsen’s [66] model assumes that attitudes toward an entity are a central factor that influences aesthetic evaluation (i.e., in the *Mind* facet).

### Limitations

Limitations that need to be kept in mind when evaluating the results of the present study include the operationalization of age. In this study, we dichotomized age into „younger than 50”and „older than 50,”a method that, while facilitating clear comparisons and supporting our theoretical framework, may oversimplify the spectrum of aesthetic appreciation across different age cohorts. The dichotomization approach assumes a homogeneity within age groups that might not exist, potentially conflating the perceptions of an 18-year-old with a 49-year-old and distinguishing these from someone just a year older. Our approach does, therefore, not minimize error variance, and is thus conservative. Future studies may benefit from considering age as a continuous variable or adopting a trichotomized model to capture more nuanced age-related differences in tattoo appreciation [100]. Further, the pragmatic dichotomization of age in our study, while simplifying statistical analyses and comparisons and serving as a very crude indicator for socialization, likely increases error variance. This could inadvertently work against our hypotheses by obscuring subtler age-related differences in aesthetic appreciation.

Moreover, Kuwahara [101] postulated that tattoos can be considered an aesthetic entity from three perspectives: (1) the tattoo itself, along with its characteristics, (2) the quality of the artistic performance, and (3) the appearance of a person’s tattooed body. Since the tattoos were only presented in connection with the bodies of the models in our design, an interaction cannot be ruled out. Such an interaction would imply that while cognitively processing the perceptually presented stimuli, the participants would not be able to differentiate between the two entities. In other words, the mental representation of the stimulus comprised an overall image of the wearer and the tattoo, which was subsequently evaluated as such.

Tattoos are a very heterogeneous phenomenon, because of the high variety in colors, styles, motifs, and tattooable body parts [10]. Our use of natural and animal motifs, as well as geometric shapes, limits the generalizability and replicability of our results [12]. Nevertheless, this study shows that, given our experimental conditions, the aesthetic appreciation of a stimulus can be influenced by tattoos. The highlighted homogeneous direction of this effect must be critically questioned and needs to be verified in replication studies. Thus, based on this study’s results, it cannot be generally assumed that perceived beauty decreases when an individual is tattooed.

Further, the expert group and the over-50 group were much smaller than their respective comparison groups. The conditions required for the hypothesis-specific statistical analyses of the data were met, but since other prerequisites were partially violated in the second part of the study, the interaction effects should be interpreted with caution.

In addition, the operationalization that was used could be questioned. For example, it remains questionable whether the Moderate condition was truly perceived as „Moderate”interindividually. Thus, in addition to the subjective experience of beauty, participants could have categorized the stimuli into the six conditions. The use of so-called nonevaluative-descriptive judgments is common in the psychology of aesthetics and is applied alongside the use of evaluative judgments [102]. Thus, performing an operationalization check would have allowed us to examine the validity of the individual tattoo conditions. That is, it would have allowed us to examine, for example, whether experts perceived the tattoos of the Extreme condition as less „Extreme” than nonexperts.

Aesthetic appreciation was operationalized via beauty ratings. In any case, the elicitation of aesthetic appreciation represents one of the central challenges of the psychology of aesthetics [103]: Aesthetic experience may be the result of an interaction between perception of the stimulus and parallel cognitive processes. Thus, appreciation cannot be isolated from the *Mind* facet [66]. It can be assumed that individuals have long-term memory representations of the schema and scripts of different aesthetic domains [103]. These background processes should be controlled, at least in part, by eliciting explicit attitudes toward tattoos. Nevertheless, implicit attitudes, as well as experiences, stereotypes, and schemas, can also influence judgments, and these can only be controlled to a very limited extent. Furthermore, the *Body* facet of aesthetic judgments [66] was not controlled in this study. This is because investigating the influences of neurological factors on aesthetic judgments requires equipment that cannot be easily used in an online setting. Nevertheless, follow-up studies could investigate the presence of neurological patterns, as these may influence aesthetic processing [104]. We also recommend that follow-up studies should investigate the differences between hetero-, homo-, bi-, and asexual individuals, because there are indications of differences in the aesthetic appreciation of tattooed individuals between sexual orientations [41].

### Further considerations

Our approach to considering age as a proxy for social norms in the appreciation of tattoos, while informed by existing literature [43–45, 72], may not fully capture the intricate ways in which social norms influence aesthetic judgments. Distinctions in appreciation across age groups may reflect a range of factors, including but not limited to evolving societal attitudes towards tattoos. We recognize the potential overlap between age-related factors and broader social views, such as varying health concerns across the lifespan that may impact perceptions of tattoos [9, 26, 105]. Future research could therefore benefit from incorporating direct assessments of social norms pertaining to tattoos to gauge the interplay more accurately between age and societal attitudes. Implementing methods to measure social norm attitudes among different age cohorts could refine our understanding of how these norms influence the aesthetic appreciation of tattoos. This refined approach would allow for a deeper investigation into the complex relationship between age, social norms, and the perception of tattoos, contributing to a more robust empirical basis for these considerations in aesthetic appreciation.

## Conclusions

Despite its limitations, the present study enriches the previously unexplored field of tattoo aesthetics and offers insights that can be further explored in subsequent studies. We have shown that tattoos influence the appreciation of human stimuli, and that this influence interacts with individuals’ internalized social norms, expertise, and tattoo status. Specifically, we found differences in perceived beauty between experts and nonexperts, tattooed and nontattooed individuals, and individuals who are older and younger than 50. Despite the limited generalizability of our results with regard to an overall negative influence of tattoos on human aesthetics, numerous implications for research and practice can be derived from the findings.

## Supporting information

S1 Table Sociodemographic characteristics of participants. The Kinsey scale was used to classify participants’ sexual orientation.
(DOCX)

S2 Table ANOVA results for the effects of expertise and condition on aesthetic appreciation. *df*_*n*_ = Degrees of Freedom numerator; *df*_*d*_ = Degrees of Freedom denominator; Epsilon (ε) represents the adjustment to the degrees of freedom for the test of Condition to account for violations of sphericity; *F* = F-ratio; *p* = significance level; *η*^*2*^ = eta squared.
(DOCX)

S3 Table Posthoc mean difference in aesthetic appreciation ratings by expertise group and tattoo condition. *Mdiff* = Mean Difference. 95%-CI = Confidence Interval. *p* = significance level.
(DOCX)

S4 Table ANOVA results for the effects of tattoo status and condition on aesthetic appreciation. *df*_*n*_ = Degrees of Freedom numerator; *df*_*d*_ = Degrees of Freedom denominator; Epsilon (ε) represents the adjustment to the degrees of freedom for the test of Condition to account for violations of sphericity; *F* = F-ratio; *p* = significance level; *η*^*2*^ = eta squared.
(DOCX)

S5 Table Posthoc mean difference in aesthetic appreciation ratings by tattoo status group and tattoo condition. *Mdiff* = Mean Difference. 95%-CI = Confidence Interval. *p* = significance level.
(DOCX)

S6 Table ANOVA results for the effects of age and condition on aesthetic appreciation. *df*_*n*_ = Degrees of Freedom numerator; *df*_*d*_ = Degrees of Freedom denominator; Epsilon (ε) represents the adjustment to the degrees of freedom for the test of Condition to account for violations of sphericity; *F* = F-ratio; *p* = significance level; *η*^*2*^ = eta squared.
(DOCX)

S7 Table Posthoc mean differences in aesthetic appreciation ratings by age group and tattoo condition. *Mdiff* = Mean Difference. 95%-CI = Confidence Interval. *p* = significance level.
(DOCX)
