# Supplementary material for: Effects of tattoos on the aesthetic appreciation of human stimuli as influenced by expertise, tattoo status, and age reflecting internalized social norms
Source: PLoS One. 2024 Dec 11;19(12):e0313940. doi: 10.1371/journal.pone.0313940 (PMC11633991; doi:10.1371/journal.pone.0313940)
Supplement: S1 Table — The Kinsey scale was used to classify participants’ sexual orientation. (DOCX) [file pone.0313940.s001.docx]

**Supporting Information 1**

**Table 1**

*Sociodemographic Characteristics of Participants*

| **Variable** | ***n*** | **%** |
| --- | --- | --- |
| **Age** |  |  |
| Younger than 50 | 409 | 84% |
| Older than 50 | 78 | 16% |
| **Tattoo Status** |  |  |
| Tattooed | 251 | 51.54% |
| Not Tattooed | 236 | 48.46% |
| **Gender Identity** |  |  |
| Male | 247 | 50.72% |
| Female | 236 | 48.46% |
| Diverse | 4 | 0.82% |
| **Sexual Orientation (Kinsey Scale)** |  |  |
| Exclusively Heterosexual | 381 | 78.23% |
| Exclusively Homosexual | 9 | 1.84% |
| Bisexual | 89 | 18.28% |
| No Sociosexual Contacts/Reactions | 8 | 1.64% |
| **Expertise as Tattoo Artist** |  |  |
| Less than 7 years | 360 | 73.92% |
| 7 years or more | 127 | 26.08% |
| **Current Occupation** |  |  |
| University Enrolled | 175 | 35.93% |
| Full-time Professional | 245 | 50.31% |
| Part-time Professional | 43 | 8.85% |

*Note. The Kinsey scale was used to classify participants’ sexual orientation.*
