# Supplementary material for: Effects of tattoos on the aesthetic appreciation of human stimuli as influenced by expertise, tattoo status, and age reflecting internalized social norms
Source: PLoS One. 2024 Dec 11;19(12):e0313940. doi: 10.1371/journal.pone.0313940 (PMC11633991; doi:10.1371/journal.pone.0313940)
Supplement: S3 Table — Mdiff = Mean Difference. 95%-CI = Confidence Interval. p = significance level. (DOCX) [file pone.0313940.s003.docx]

**Supporting Information 2**

**Table 3**

*Posthoc Mean Difference in Aesthetic Appreciation Ratings by Expertise Group and Tattoo Condition*

| Variable | Condition | *Mdiff* | 95%-CI | *p* |
| --- | --- | --- | --- | --- |
| Experts vs. Nonexperts |  |  |  |  |
|  | Baseline | -0.20 | -0.02, 0.42 | 1.00 |
|  | Light | -0.14 | -0.08, 0.36 | 1.00 |
|  | Moderate | 0.28 | -0.50, -0.05 | .99 |
|  | Heavy | 0.40 | -0.62, -0.18 | .03 |
|  | Extreme | -1.36 | -1.59, -1.14 | < .001 |
|  | Extreme + Face | -1.13 | -1.35, -0.90 | <. 001 |
| Experts |  |  |  |  |
|  | Baseline - Light | 0.03 | -0.14, 0.21 | 1.00 |
|  | Light - Moderate | 0.21 | 0.03, 0.38 | 1.00 |
|  | Moderate - Heavy | 0.03 | -0.15, 0.21 | 1.00 |
|  | Heavy - Extreme | -0.54 | -0.72, -0.36 | < .001 |
|  | Extreme – Extreme + Face | 0.80 | 0.62, 0.98 | < .001 |
| Nonexperts |  |  |  |  |
|  | Baseline - Light | 0.09 | -0.01, 0.20 | 1.00 |
|  | Light - Moderate | 0.63 | 0.52, 0.73 | < .001 |
|  | Moderate - Heavy | -0.15 | 0.05, 0.26 | .28 |
|  | Heavy - Extreme | 0.42 | 0.32, 0.53 | < .001 |
|  | Extreme – Extreme + Face | 0.57 | -0.46, 0.67 | < .001 |

*Note. Mdiff* = Mean Difference. 95%-CI = Confidence Interval. *p* = significance level
