# Supplementary material for: Effects of tattoos on the aesthetic appreciation of human stimuli as influenced by expertise, tattoo status, and age reflecting internalized social norms
Source: PLoS One. 2024 Dec 11;19(12):e0313940. doi: 10.1371/journal.pone.0313940 (PMC11633991; doi:10.1371/journal.pone.0313940)
Supplement: S5 Table — Mdiff = Mean Difference. 95%-CI = Confidence Interval. p = significance level. (DOCX) [file pone.0313940.s005.docx]

**Supporting Information 2**

**Table 5**

*Posthoc Mean Difference in Aesthetic Appreciation Ratings by Tattoo Status Group and Tattoo Condition*

| Variable | Condition | *Mdiff* | 95%-CI | *p* |
| --- | --- | --- | --- | --- |
| Tattooed vs. Nontattooed |  |  |  |  |
|  | Baseline | 0.35 | 0.15, 0.54 | .03 |
|  | Light | 0.22 | 0.02, 0.41 | 1.00 |
|  | Moderate | -0.27 | -0.46, -0.07 | .44 |
|  | Heavy | -0.43 | -0.62, 0.24 | < .001 |
|  | Extreme | -1.38 | -1.60, -1.19 | < .001 |
|  | Extreme + Face | -1.04 | -1.23, -0.84 | <. 001 |
| Tattooed |  |  |  |  |
|  | Baseline - Light | 0.01 | -0.11, 0.14 | 1.00 |
|  | Light - Moderate | 0.28 | 0.16, 0.40 | < .001 |
|  | Moderate - Heavy | 0.04 | -0.08, 0.16 | 1.00 |
|  | Heavy - Extreme | -0.29 | -0.41, -0.17 | < .001 |
|  | Extreme – Extreme + Face | 0.80 | 0.67, 0.92 | < .001 |
| Nontattooed |  |  |  |  |
|  | Baseline - Light | 0.14 | 0.19, 0.27 | 1.00 |
|  | Light - Moderate | 0.77 | 0.62, 0.89 | < .001 |
|  | Moderate - Heavy | 0.21 | 0.08, 0.33 | .09 |
|  | Heavy - Extreme | 0.66 | 0.53, 0.79 | < .001 |
|  | Extreme – Extreme + Face | 0.45 | 0.33, 0.58 | < .001 |

*Note. Mdiff* = Mean Difference. 95%-CI = Confidence Interval. *p* = significance level
