# Supplementary material for: Effects of tattoos on the aesthetic appreciation of human stimuli as influenced by expertise, tattoo status, and age reflecting internalized social norms
Source: PLoS One. 2024 Dec 11;19(12):e0313940. doi: 10.1371/journal.pone.0313940 (PMC11633991; doi:10.1371/journal.pone.0313940)
Supplement: S6 Table — dfn = Degrees of Freedom numerator; dfd = Degrees of Freedom denominator; Epsilon (ε) represents the adjustment to the degrees of freedom for the test of Condition to account for violations of sphericity; F = F-ratio; p = significance level; η2 = eta squared. (DOCX) [file pone.0313940.s006.docx]

**Supporting Information 2**

**Table 6**

*ANOVA Results for the Effects of Age and Condition on Aesthetic Appreciation*

| Predictor | *df_n_* | *df_d_* | ε | *F* | *p* | *η^2^* |
| --- | --- | --- | --- | --- | --- | --- |
| Age | 1 | 485 | .00 | 5.20 | .023 | .007 |
| Condition | 5 | 2425 | .35 | 260.39 | < .001 | .17 |
| Age:Condition | 5 | 2425 | .35 | 8.97 | < .001 | .007 |

*Note. df_n_* = Degrees of Freedom numerator; *df_d_* = Degrees of Freedom denominator; Epsilon (ε) represents the adjustment to the degrees of freedom for the test of Condition to account for violations of sphericity; *F* = F-ratio; *p* = significance level; *η^2^* = eta squared.
