# Supplementary material for: Effects of tattoos on the aesthetic appreciation of human stimuli as influenced by expertise, tattoo status, and age reflecting internalized social norms
Source: PLoS One. 2024 Dec 11;19(12):e0313940. doi: 10.1371/journal.pone.0313940 (PMC11633991; doi:10.1371/journal.pone.0313940)
Supplement: S7 Table — Mdiff = Mean Difference. 95%-CI = Confidence Interval. p = significance level. (DOCX) [file pone.0313940.s007.docx]

**Supporting Information 2**

**Table 7**

*Posthoc Mean Differences in Aesthetic Appreciation Ratings by Age Group and Tattoo Condition*

| Variable | Condition | *Mdiff* | 95%-CI | *p* |
| --- | --- | --- | --- | --- |
| <50 vs. >50 |  |  |  |  |
|  | Baseline | -0.06 | -0.34, 0.21 | 1.00 |
|  | Light | -0.01 | -0.29, 0.26 | 1.00 |
|  | Moderate | 0.20 | -0.08, 0.48 | 1.00 |
|  | Heavy | 0.28 | 0.004, 0.56 | 1.00 |
|  | Extreme | 0.70 | 0.43, 0.98 | < .001 |
|  | Extreme + Face | 0.42 | -0.14, 0.69 | .21 |
| <50 |  |  |  |  |
|  | Baseline - Light | 0.07 | -0.03, 0.17 | 1.00 |
|  | Light - Moderate | 0.48 | 0.38, 0.59 | < .001 |
|  | Moderate - Heavy | 0.11 | 0.03, 0.21 | 1.00 |
|  | Heavy - Extreme | 0.10 | -0.0004, 0.21 | 1.00 |
|  | Extreme – Extreme + Face | 0.68 | 0.57, 0.78 | < .001 |
| >50 |  |  |  |  |
|  | Baseline - Light | -.12 | 0.12, 0.36 | 1.00 |
|  | Light - Moderate | .70 | 0.46, 0.94 | < .001 |
|  | Moderate - Heavy | .19 | -0.05, 0.43 | 1.00 |
|  | Heavy - Extreme | .53 | 0.29, 0.77 | < .001 |
|  | Extreme – Extreme + Face | .39 | 0.15, 0.63 | .09 |

*Note. Mdiff* = Mean Difference. 95%-CI = Confidence Interval. *p* = significance level
